# Hypoxia with Wharton’s jelly mesenchymal stem cell coculture maintains stemness of umbilical cord blood-derived CD34^+^ cells

**DOI:** 10.1186/s13287-018-0902-5

**Published:** 2018-06-13

**Authors:** Dewan Zhao, Lingjia Liu, Qiang Chen, Fangfang Wang, Qiuyang Li, Qiang Zeng, Jingcao Huang, Maowen Luo, Wenxian Li, Yuhuan Zheng, Ting Liu

**Affiliations:** 1 Department of Hematology, Hematology Research Laboratory, West China Hospital of Sichuan University, Chengdu, Sichuan People’s Republic of China; 2 Sichuan Cord Blood Stem Cell Bank, Chengdu, Sichuan People’s Republic of China

**Keywords:** Hypoxic coculture, Wharton’s jelly mesenchymal stem cells, Ex vivo HSPC expansion

## Abstract

**Background:**

The physiological approach suggests that an environment associating mesenchymal stromal cells with low O_2_ concentration would be most favorable for the maintenance of hematopoietic stem/progenitor cells (HSPCs). To test this hypothesis, we performed a coculture of cord blood CD34^+^ cells with Wharton’s jelly mesenchymal stem cells (WJ-MSCs) under different O_2_ concentration to simulate the growth of HSPCs in vivo, and assessed the impacts on stemness maintenance and proliferation of cord blood HSPCs in vitro.

**Methods:**

CD34^+^ cells derived from cord blood were isolated and cocultured under 1%, 3%, or 20% O_2_ concentrations with irradiated WJ-MSCs without adding exogenous cytokines for 7 days. The cultured cells were harvested and analyzed for phenotype and functionality, including total nuclear cells (TNC), CD34^+^Lin^−^ cells, colony forming unit (CFU) for committed progenitors, and long-term culture initiating cells (LTC-ICs) for HSPCs. The cytokine levels in the medium were detected with Luminex liquid chips, and the mRNA expression of hypoxia inducible factor (HIF) genes and stem cell signal pathway (Notch, Hedgehog, and Wnt/β-catenin) downstream genes in cord blood HSPCs were confirmed by quantitative real-time polymerase chain reaction (qRT-PCR).

**Results:**

Our results showed that the number of TNC cells, CD34^+^Lin^−^ cells, and CFU were higher or similar with 20% O_2_ (normoxia) in coculture and compared with 1% O_2_ (hypoxia). Interestingly, a 1% O_2_ concentration ensured better percentages of CD34^+^Lin^−^ cells and LTC-IC cells. The hypoxia tension (1% O_2_) significantly increased vascular endothelial growth factor (VEGF) secretion and decreased interleukin (IL)-6, IL-7, stem cell factor (SCF), and thrombopoietin (TPO) secretion of WJ-MSCs, and selectively activated the Notch, Wnt/β-catenin, and Hedgehog signaling pathway of cord blood HSPCs by HIF-related factors, which may play an important role in stemness preservation and for sustaining HSPC quiescence.

**Conclusions:**

Our data demonstrate that cord blood HSPCs maintain stemness better under hypoxia than normoxia with WJ-MSC coculture, partially due to the increased secretion of VEGF, decreased secretion of IL-6 by WJ-MSCs, and selective activation of stem cell signal pathways in HSPCs. This suggests that the oxygenation may not only be a physiological regulatory factor but also a cell engineering tool in HSPC research, and this may have important translational and clinical implications.

## Background

Umbilical cord blood (UCB) is an alternative source of hematopoietic stem/progenitor cells (HSPCs) for transplantation in malignant and nonmalignant hematologic diseases. However, since the amount of HSPCs in a single unit of cord blood is insufficient for transplantation in most adult patients, the application of cord blood HSPCs remains with a major limitation [1]. The ex-vivo expansion of cord blood HSPCs is one feasible method to increase the HSPC number and has recently become a focus for research. HSPCs maintain their stemness by interacting with stromal cells and the extracellular matrix through cell-to-cell contact and paracrine factor secretion [2]. The microenvironment of the placenta or umbilical cord, where the UCB-HSPCs reside in, is different to that in bone marrow, with the stromal cells including Wharton’s jelly mesenchymal stem cells (WJ-MSCs) and vascular endothelial cells being dissimilar to bone marrow stromal cells (e.g., no osteoblasts). Compared with MSCs from the bone marrow, WJ-MSCs not only express cell markers of BM-MSCs, but also additionally express many molecules involved in HSPC expansion and interaction, such as granulocyte colony stimulating factor (G-CSF), granulocyte macrophage colony stimulating factor (GM-CSF), and CD117 [3]. These advantages make WJ-MSCs a preferable feeder layer choice for UCB-HSPC expansion in vitro. Furthermore, in physiological situations, HSPCs are distributed in the tissues with low oxygen tension, and it is now widely accepted that gradients of oxygen from below 1% in hypoxic niches to 6% in the sinusoidal cavity exist within the human bone marrow [4, 5]. Like all other established cell lines, stem cells were typically cultured under ambient oxygen tension with very little attention paid to the metabolic milieu of the niche in which they were grown or normally resided. In this study, we compared the effects of combining WJ-MSCs at different O_2_ concentrations in coculture on stemness maintenance and proliferation of HSPCs in vitro without adding exogenous cytokines. Our results show that stemness of HPSCs can be better maintained at 1% O_2_ with WJ-MSC coculture. Under hypoxia, the levels of secretion of different cytokines via WJ-MSCs and selective activation of stem cell signal pathways may impact a few mechanisms.

## Methods

### Isolation and characterization of WJ-MSCs from umbilical cord

**Fig. 1:**
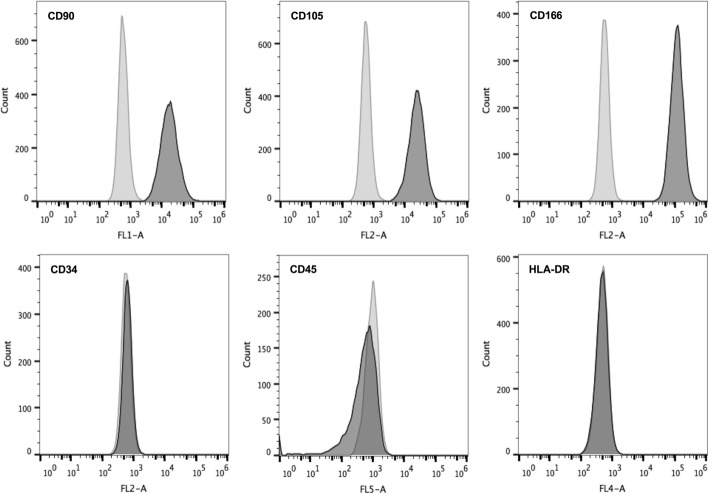
The characterization of WJ-MSCs. Wharton’s jelly mesenchymal stem cells (MSCs) were positive for CD90, CD105, and CD166, and negative for CD34, CD45, and HLA-DR. The light gray histograms show the isotype control-stained cells and the dark gray histograms show the antibody-stained cells. The control population was stained with matched isotype antibodies

Umbilical cord samples were collected from healthy full-term deliveries after obtaining informed consent as a donation for research. The preparation of the umbilical cord tissue was completed within 12 h. The umbilical cord sample was rinsed with normal saline to remove the residual blood. It was then cut into 1.0-cm long segments, and each segment was cut into a 1.0 mm^3^ tissue mass and evenly plated in a 10.0-cm dish. The dishes were incubated at 37 °C under 5% CO_2_ for 24 h, and then 10 ml culture medium contained 45% low-glucose Dulbecco’s modified Eagle’s medium (DMEM), 45% Ham’s F-12, and 10% fetal bovine serum (FBS) (Life Technologies, Grand Island, NY, USA) was added. Half of the medium was replaced 7 days later, and then half of the medium was replaced every 3 days until the primary cells reached subconfluence. The primary cells were detached with trypsin-EDTA (0.25%) (HyClone Laboratories, Logan, Utah, USA) and the reaction was terminated with FBS to passage. Cells harvested from every three dishes were plated in a T-75 flask, and daughter cells were passaged at 1:4 ratios in T-75 flasks. The phenotypic characterization on second- to fourth-passage WJ-MSCs was assayed using a FACScan flow cytometer (Beckman Coulter, USA) for CD90-FITC, CD45-PECY7 (eBioscience, San Diego, CA, USA), CD105-PE, HLA-DR-PECY5, CD34-PE, and CD166-PE (Biolegend, San Diego, CA, USA) according to the manufacturer’s instructions. Appropriate isotype controls for nonspecific binding were set for each antibody. For each sample, the assayed cells were analyzed for at least 10,000 events. The WJ-MSCs expressed CD90, CD105, and CD166, and were negative for CD45, CD34, and HLA-DR (Fig. 1).

### Isolation and purification of CD34^+^ cells from umbilical cord blood

Umbilical cord blood was collected from normal full-term delivery after obtaining informed consent from the mothers as a donation for banking, and only cord blood samples not appropriate for banking (< 100 ml) were used in our experiments. We mixed cord blood with 6% hetastarch in 0.9% sodium chloride (Hospira, USA) at a ratio of 4:1 and let it stand for approximately 30 min to allow most of the red cells to form a sediment. Cells in the supernatant was laid onto Ficoll-Paque PLUS (GE Healthcare Bio-Sciences, Pittsburgh, USA) and centrifuged to collect mononuclear cells (MNCs) by depleting the platelets, plasma, and residual red cells. Enrichment of CD34^+^ cells was performed with two runs of immunomagnetic selection on MiniMACS columns (Miltenyi Biotec, Gladbach, Germany) in accordance with the manufacturer’s instructions.

### Coculture of WJ-MSCs and CD34^+^ cells under hypoxic or normoxic conditions

WJ-MSCs at passages 2 to 4 were harvested and radiated (25 Gy) to prevent overgrowth. We then plated them into 12-well plates (8 × 10^4^/well) with H5100 medium contained 10^−6^ M hydrocortisone (StemCell Technologies, Vancouver, BC, Canada). At least 24 h later, we removed the medium and seeded purified CD34^+^ cells (suspended in H5100 medium with 10^−6^ M hydrocortisone) into the plate (4 × 10^4^/well). WJ-MSCs and CD34^+^ cells were cocultured at 37 °C and 5% CO_2_ under normoxic (20% O_2_) and hypoxic (3% and 1% O_2_) conditions for 7 days without adding exogenous cytokines, replacing half of the medium every 3 days. Hypoxic cultures were made in a two-gas incubator (Thermo Scientific, Forma™ Steri-Cycle i160 STERI-cycle, USA) equipped with an O_2_ probe to regulate N_2_ levels. We also set a control group without WJ-MSCs as feeder layers, with the medium and culture conditions as described above.

### Flow cytometry analysis

We defined the CD34^+^, CD10^−^, CD14^−^, and CD19^−^ cells as CD34^+^Linage^−^ (CD34^+^Lin^−^) cells, which represent cord blood HSPCs. At day 7, we collected the supernatant and detached the adherent cells containing WJ-MSCs and HSPCs (attached to the WJ-MSCs). Viable total nucleated cells (TNC) were counted via the Trypan Blue staining method. Flow cytometry staining was performed with CD34-PECY7, CD45-FITC (eBioscience, San Diego, CA, USA), CD10-APC, CD14-APC/Cy7, and CD19-PE (Biolegend, San Diego, CA, USA) antibodies, and then samples were analyzed using a FACScan flow cytometer (Beckman Coulter, USA). For each sample, at least 30,000 events were recorded. The isotype antibodies were used to determine the level of nonspecific binding.

### Colony-forming cell assay

On day 7, the harvested cells from each group were plated in semisolid culture (H4434, Stem Cell Technologies, Vancouver, BC, Canada) following the manufacturer’s instructions for the colony-forming unit (CFU) assay. After incubation at 37 °C under 5% CO_2_ at 100% humidity for 14 days, the total colony-forming unit (T-CFU), burst-forming unit-erythroid (BFU-E), and colony-forming unit-granulocyte/macrophage (CFU-GM) levels were scored under an inverted microscope. Each CFU is equivalent to a colony-forming cell (CFC).

### Long-term culture-initiating cell assay

M2-10B4 (ATCC), a murine fibroblast cell line, was used as a feeder layer. At least 24 h before assay, M2-10B4 cells were radiated (80 Gy) and plated in six-well plates (2.5 × 10^5^/well). The plates were coated with collagen solution (StemCell Technologies, Vancouver, BC, Canada). Cells harvested from coculture systems at different oxygen concentrations on day 7 were resuspended with H5100 containing 10^−6^ M hydrocortisone (StemCell Technologies, Vancouver, BC, Canada) and then seeded into the plate (2 × 10^5^/well) with the feeder layers. At weekly intervals we replaced half of the medium. Both nonadherent and adherent cells were harvested at week 5 and plated in semisolid culture (H4434, StemCell Technologies, Vancouver, BC, Canada) for CFC assay. After 18 days, colonies were scored under an inverted microscope. The long-term culture-initiating cell (LTC-IC) number was calculated according to the manufacturer’s instructions.

### Cytokine concentration analysis

WJ-MSCs were radiated (25 Gy) and plated into 12-well plates (8 × 10^4^/well) suspended with H5100 medium containing 10^−6^ M hydrocortisone (StemCell Technologies, Vancouver, BC, Canada). Twenty-four hours later, we replaced the medium and cultured the WJ-MSCs at 20% O_2_ or 1% O_2_, replacing half of the medium twice a week. On day 7, we collected the supernatant of the medium and analyzed the concentration of the following cytokines in each group by Luminex assays Kit (R&D Systems, USA) on Luminex 200: tumor necrosis factor (TNF)-alpha, interleukin (IL)-6, IL-3, vascular endothelial growth factor (VEGF), stem cell factor (SCF), IL-7, GM-CSF, macrophage colony stimulating factor (M-CSF), G-CSF, and thrombopoietin (TPO). H5100 culture medium with 10^−6^ M hydrocortisone incubated under the same conditions for each group was set as a blank control.

### Signal pathway assays in MSC-HSPC low O_2_ cocultures

We harvested the nonadherent cells of each coculture system on day 7. Total RNA was isolated using the MicroElute Total RNA Kit (OMEGA Bio-tek, Norcross, Georgia). Single-strand cDNA was synthesized using the HiFiScript cDNA Synthesis Kit (CW-Bio, Jiangsu, China). Quantitative real-time polymerase chain reaction (qRT-PCR) was performed using the SYBR Green PCR Master Mix (Fermentas, Vilnius, Lithuania) on a CFX96 Touch™ Real-Time PCR Detection System (Bio-Rad). The housekeeping gene β-actin was used as a reference gene. Detected genes included: the hypoxia inducible genes HIF1-α, HIF2-α, and ARNT; the Notch pathway downstream genes HES1, HES3, HEY1, and HEY2; the Wnt/β-catenin downstream genes AXIN2, MMP7, and TCF-1; and the Hedgehog downstream genes GLI1, PTCH1, PTCH2, and SMO. Gene-specific primer sets are listed in Table 1.

**Table 1:** Quantitative real-time polymerase chain reaction primer list

Function	Gene	Forward primer	Reverse primer
Hypoxia inducible	HIF1A	GGTCTAGGAAACTCAAAACCTGA	TCCTCACACGCAAATAGCTGA
	HIF2A	ATCAGCTTCCTGCGAACACA	GCTCCACCTGTGTAAGTCCC
	ARNT	ACTACTGCCAACCCCGAAAT	CTCTGGACAATGGCTCCTCC
Notch pathway	HES1	GTGTCAACACGACACCGGAT	GGAATGCCGCGAGCTATCTT
	HES3	GATTTCCAAGCCGCTGATGG	TTCCGGATCTGGTGCGAGTA
	HEY1	TGCGGATTGAGCTAGTGCAT	AAGTAACCTTGGTCTCCCGT
	HEY2	GTGGGAAAGAGCCGCTAGG	GAGCTAGTACTTTGCCCCGA
Wnt/βcatenin pathway	AXIN2	GCAACTCAGTAACAGCCCGA	CTCCTCTCTTTTACAGCAGGGC
	MMP7	GTCTCTGGACGGCAGCTATG	GATAGTCCTGAGCCTGTTCCC
	TCF-1	CCAAGAATCCACCACAGAGACA	CAATGCCTATGGCTTCCTTGC
Hedgehog pathway	GLI1	AGCCTTCAGCAATGCCAGTGAC	GTCAGGACCATGCACTGTCTTG
	PTCH1	GCTGCACTACTTCAGAGACTGG	CACCAGGAGTTTGTAGGCAAGG
	PTCH2	GCACTATTACCGCAACTGGCTAC	TCTCCAGTCTGGATGAGCAGCT
	SMO	AATGCGTGCTTCTTTGTGGG	TCTCATTGGAGGTGGGCTCC
House keeping	β-actin	AGAGCTACGAGCTGCCTGAC	AGCACTGTGTTGGCGTACAG

### Statistical analysis

The statistical differences between each group were analyzed using the SPSS 20.0 statistical software for all the experiment data. The comparison was analyzed between two groups with an independent sample *t* test, and among three groups with single-factor analysis of variance (ANOVA). The values were plotted as mean ± standard deviation. Probability values *P* < 0.05 were considered statistically significant.

## Results

### The influence of different oxygen concentrations on cord blood HSPC coculture

**Fig. 2:**
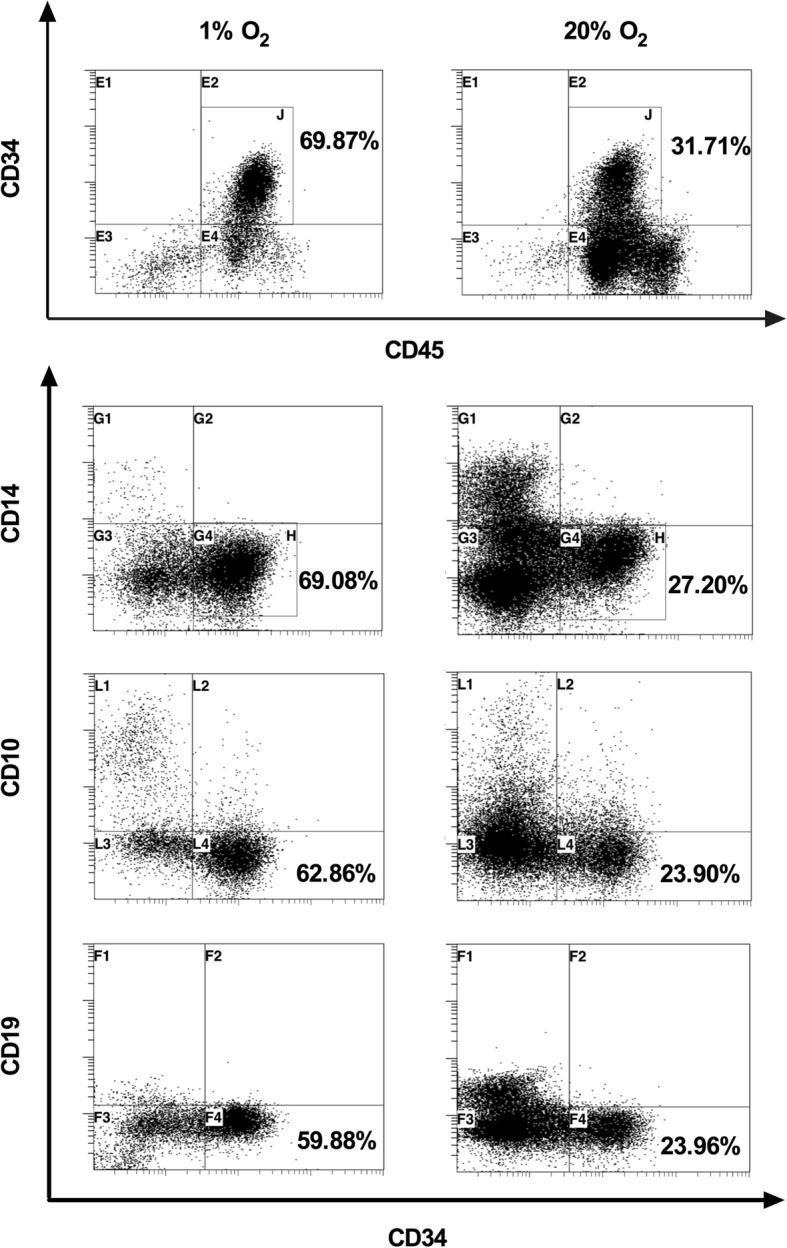
Flow cytometry analysis of 1% and 20% O_2_ coculture groups. CD45, leukocyte marker; CD34, HSPC marker; CD10, pre-B cell marker; CD14, myeloid cell marker; CD19, B cell marker. The CD34^+^CD10^−^CD14^−^CD19^−^ cells represent the CD34^+^Lin^−^ HSPC cells

**Fig. 3:**
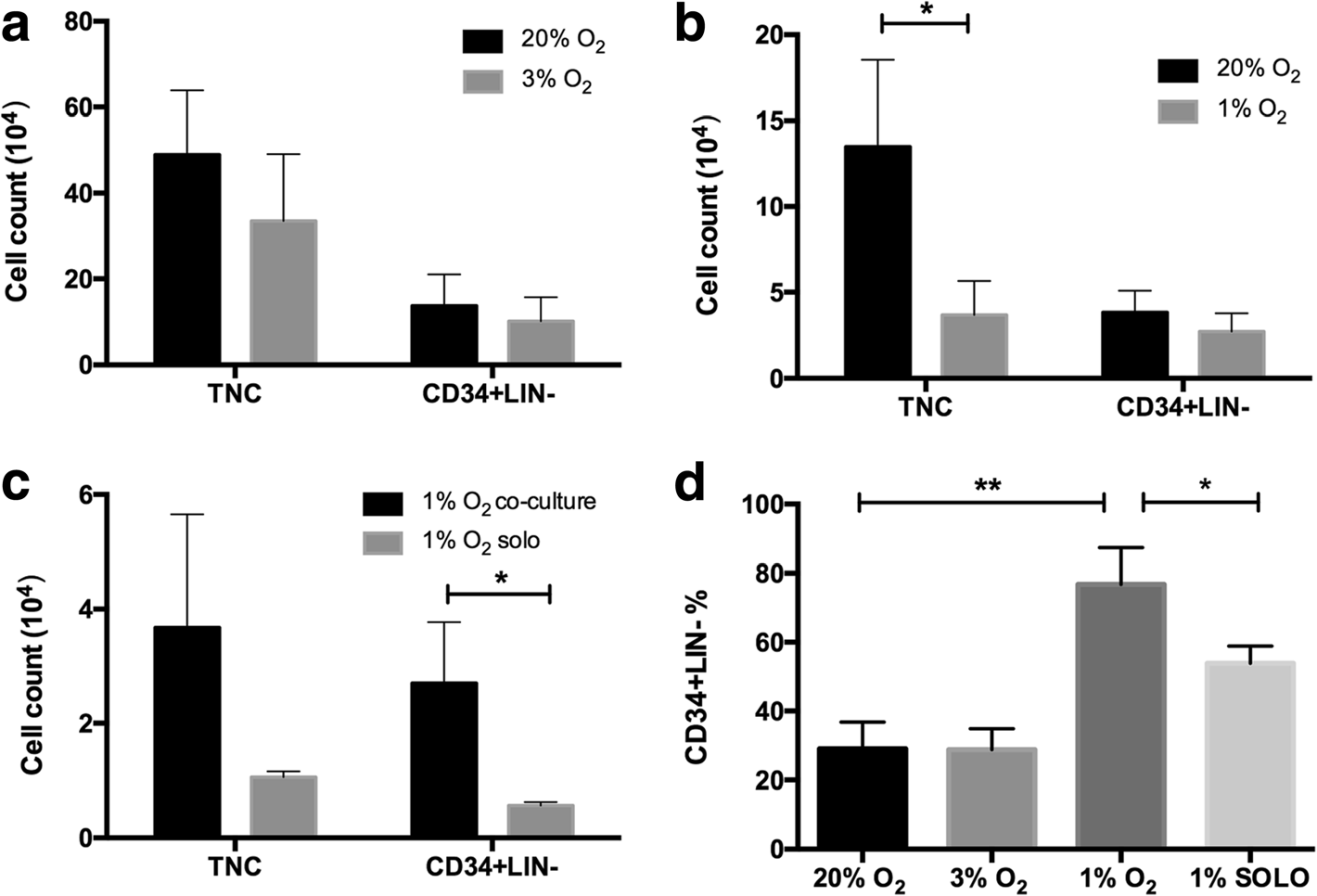
The influence of different oxygen concentration on the ex-vivo culture of cord blood HSPCs (*n* = 4). **a** The total nucleated cells (TNCs) and CD34^+^Lin^−^ cell numbers in 3% and 20% O_2_ groups on day 7. **b** The TNCs and CD34^+^Lin^−^ cell number in 1% and 20% O_2_ groups. **c** The TNCs and CD34^+^Lin^−^ cell number in coculture and suspended culture group (solo) at 1% O_2_. **d** The percentage of CD34^+^Lin^−^ cells in the different groups. *P < 0.05, **P < 0.01

In this study, we defined CD34^+^CD10^−^CD14^−^CD19^−^ phenotypic cells as the CD34^+^lin^−^ HSPC cells (Fig. 2). First, we compared the effects of 3% O_2_ with 20% O_2_ on the WJ-MSC-HSPC coculture in vitro for 7 days. The results showed that there was no significant difference between these two groups in the percentage or cell numbers of CD34^+^Lin^−^ cells, as well as the number of TNC (*P* > 0.05). We then cocultured UCB-HSPCs with WJ-MSCs at 1% O_2_ and 20% O_2_ concentrations. We collected cells on day 7, and the TNC counts at 1% O_2_ and 20% O_2_ were 3.68 ± 1.97 × 10^4^ and 13.50 ± 5.04 × 10^4^ (*P* < 0.05), respectively; the number of CD34^+^Lin^−^ cells was 2.70 ± 1.07 × 10^4^ and 3.82 ± 1.28 × 10^4^ (*P* > 0.05), respectively; and the percentage of CD34^+^Lin^−^ cells was 76.83 ± 10.56% and 29.05 ± 7.74% (*P* < 0.01), respectively. As a control, compared with the coculture group under 1% O_2_ conditions, the number of TNC and CD34^+^Lin^−^ cell in the suspension culture group was 1.06 ± 0.10 × 10^4^ and 0.57 ± 0.06 × 10^4^, respectively, and the percentage of CD34^+^Lin^−^ was 53.87 ± 5.02% (*P* < 0.05) (Fig. 3).

### Differentiation and long-term reconstruction capacity maintenance

**Fig. 4:**
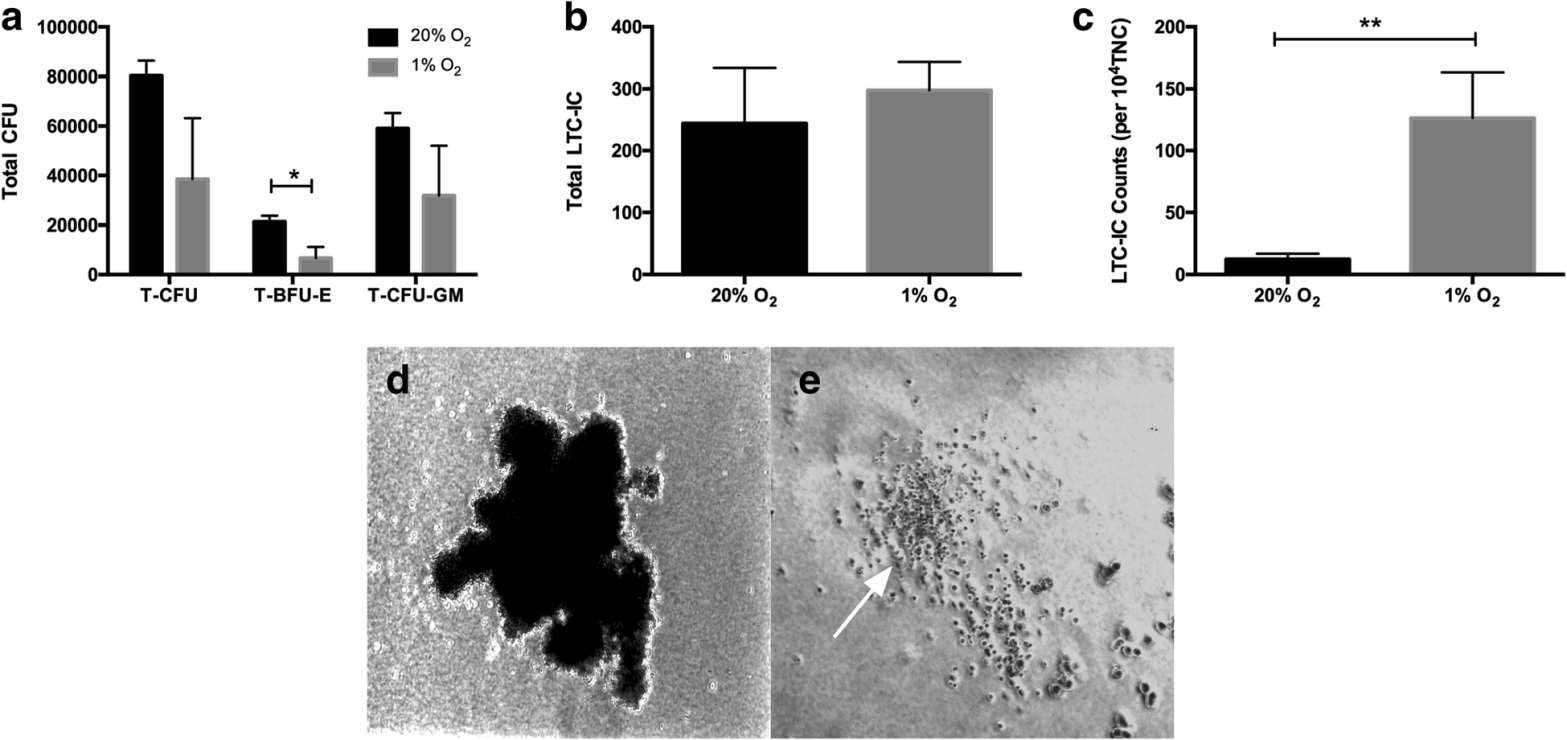
The differentiation potential and long-term reconstruction capacity maintenance of HSPCs (*n* = 4). **a** The total colony-forming unit (T-CFU), total burst-forming unit-erythroid (T-BFU-E), and total colony-forming unit-granulocyte/macrophage (T-CFU-GM) number per well in 20% and 1% O_2_ groups. **b** The long-term culture-initiating cell (LTC-IC) amount per well in 20% and 1% O_2_ groups. **c** The LTC-IC amount per 10,000 total nucleated cells (TNC) in 20% and 1% O_2_ groups. **d** An image of BFU-E. **e** An image of CFU-GM (white arrow). *P < 0.05, **P < 0.01

The TNCs were harvested on day 7 from the 1% O_2_ and 20% O_2_ culture systems for CFU and LTC-IC assay. Methylcellulose colony-forming assay was performed to evaluate the differentiation potential (CFU), and the LTC-IC was evaluated for the maintenance capacity of HSPCs in vitro (Fig. 4). Among the TNCs harvested per well, the 1% O_2_ group induced 38,542 ± 24,647 T-CFUs and 31,846 ± 20,165 CFU-GMs, and the 20% O_2_ group induced 80,331 ± 6079 T-CFUs and 59,013 ± 6283 CFU-GMs (*P* > 0.05). The BFU-E results for the 1% O_2_ and 20% O_2_ group were 6697 ± 4548 and 21,317 ± 2424 (*P* < 0.05) (Fig. 4a). The number of LTC-IC from the 1% O_2_ and 20% O_2_ groups were 298 ± 45 and 244 ± 89, respectively (*P* > 0.05). In each 10,000 TNCs, the LTC-IC amounts for the 1% O_2_ and 20% O_2_ groups were 126 ± 37 and 12 ± 4, respectively, which indicated that hypoxia culture could maintain LTC-IC count capacity better than normoxia culture (*P* < 0.05).

### Concentration of cytokines secreted by WJ-MSCs

**Fig. 5:**
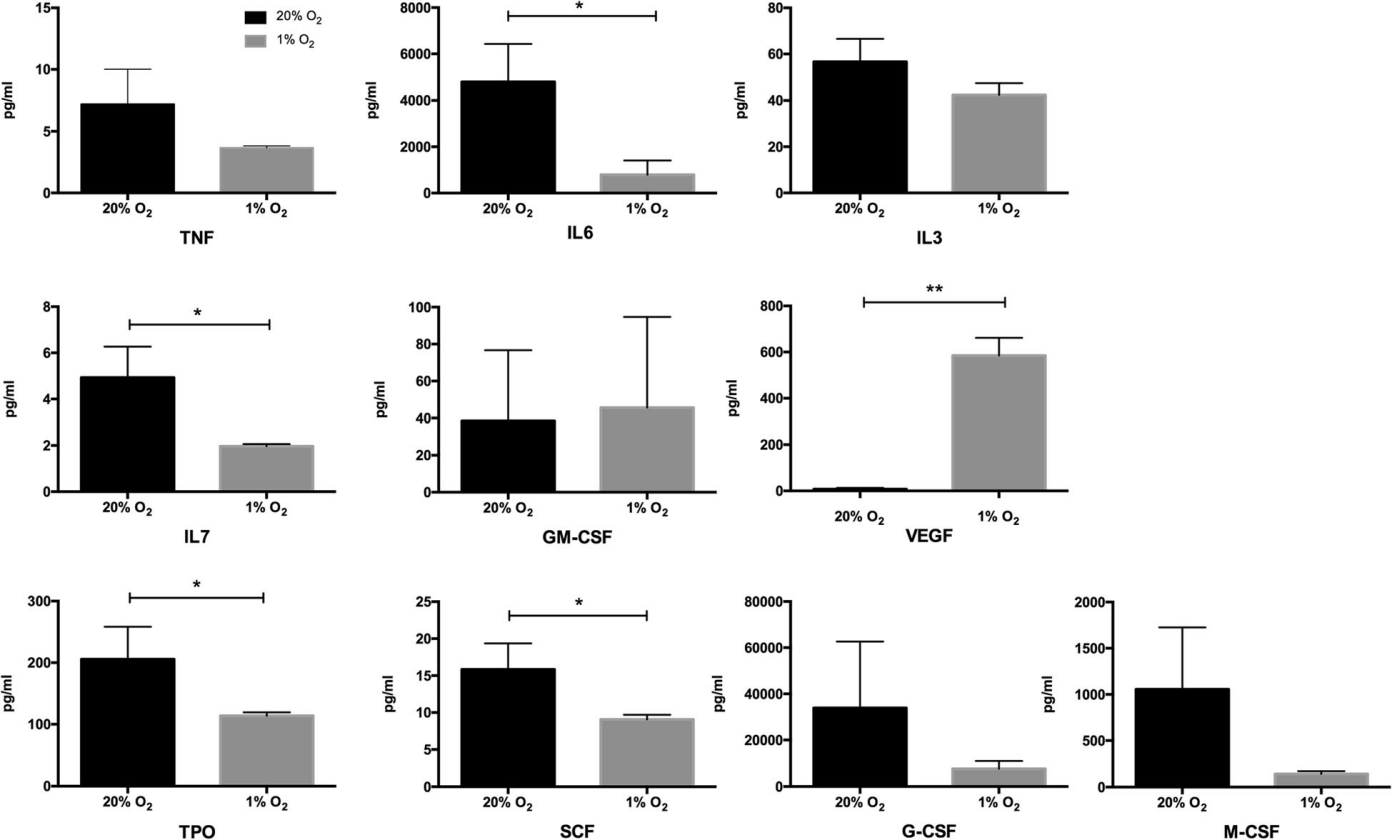
Comparison of cytokine levels in 20% and 1% O_2_ groups (*n* = 4). *P < 0.05, **P < 0.01. G-CSF granulocyte colony stimulating factor, GM-CSF granulocyte macrophage colony stimulating factor, IL interleukin, M-CSF macrophage colony stimulating factor, SCF stem cell factor, TNF tumor necrosis factor, TPO thrombopoietin, VEGF vascular endothelial growth factor

To explore the cytokine levels secreted by WJ-MSCs at different oxygen concentration, WJ-MSCs were cultured for 7 days under conditions of 1% O_2_ and 20% O_2_ and the medium was collected and the cytokines measured by Luminex liquid-phase chip. We assayed 10 cytokines related to hematopoiesis: TNF-alpha, IL-6, IL-3, VEGF, SCF, IL-7, GM-CSF, M-CSF, G-CSF, and TPO. The results show that the culture medium contained much higher levels of VEGF in the 1% O_2_ group than the 20% O_2_ group (*P* < 0.01), and more IL-6, IL-7, SCF, and TPO in the 20% O_2_ group than in the 1% O_2_ group (*P* < 0.05) (Table 2 and Fig. 5).

**Table 2:** The cytokine levels at different O_2_ tension (*n* = 4)

pg/ml	20% O_2_	1% O_2_
TNF-α	7.18 ± 2.82	3.65 ± 0.17
IL6	4789.67 ± 1642.70*	792.67 ± 615.10
IL3	56.62 ± 9.92	42.42 ± 4.95
VEGF	7.83 ± 5.29**	584.83 ± 77.11
SCF	15.82 ± 3.52*	9.08 ± 0.62
IL7	4.93 ± 1.34*	1.97 ± 0.10
GM-CSF	38.47 ± 38.23	45.65 ± 49.07
M-CSF	1056.17 ± 669.73	143.00 ± 28.99
G-CSF	33,893.83 ± 28,728.97	7725.50 ± 3391.86
TPO	205.50 ± 52.79*	113.83 ± 5.58

*G-CSF* granulocyte colony stimulating factor, *GM-CSF* granulocyte macrophage colony stimulating factor, *IL* interleukin, *M-CSF* macrophage colony stimulating factor, *SCF* stem cell factor, *TNF* Tumor necrosis factor, *TPO* thrombopoietin, *VEGF* vascular endothelial growth factor*P < 0.05, **P < 0.01, versus 1% O_2_

### Stem cell pathway activation under hypoxia

**Fig. 6:**
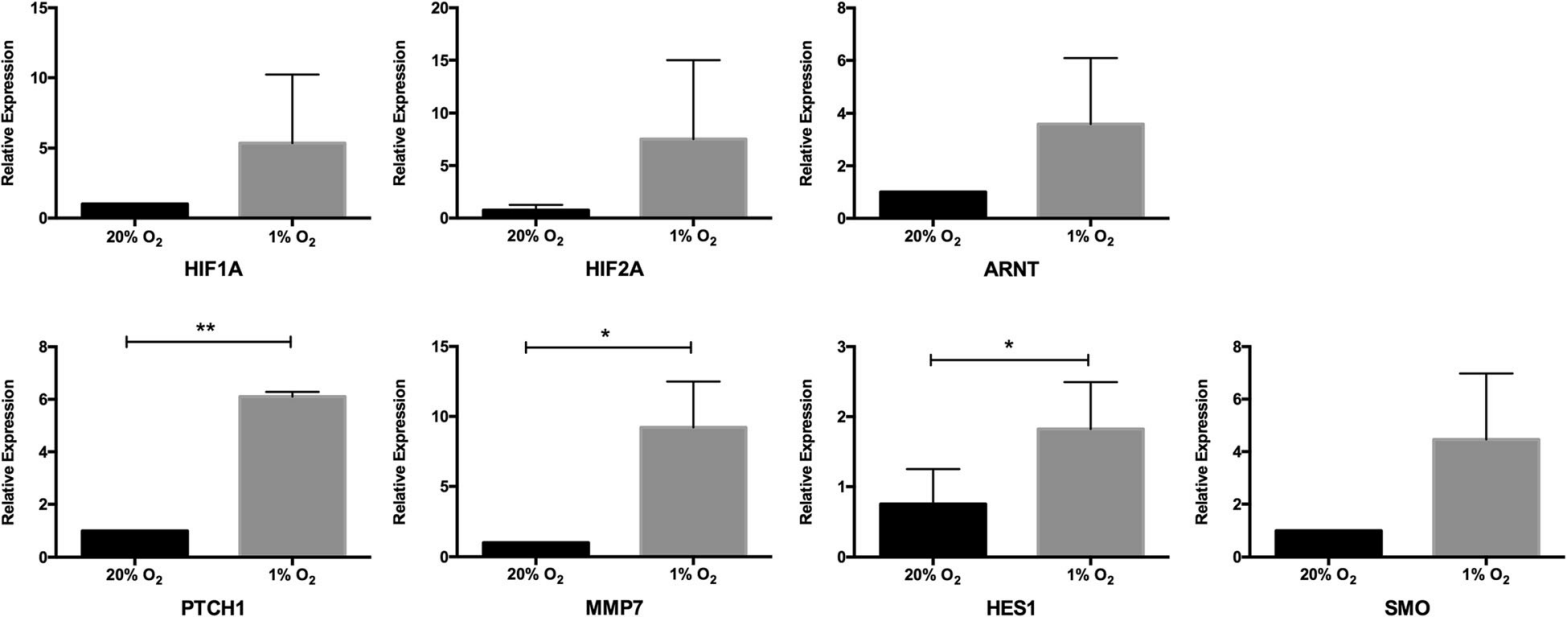
The expression levels of stem cell pathway target genes at 20% and 1% O_2_ (*n* = 4). Detected genes included hypoxia inducible genes HIF1-α, HIF2-α, and ARNT, Notch pathway downstream genes HES1, HES3, HEY1, and HEY2, Wnt/β-catenin downstream genes AXIN2, MMP7, and TCF-1, and Hedgehog downstream genes GLI1, PTCH1, PTCH2, and SMO. *P < 0.05, **P < 0.01

To investigate whether stem cell pathways were activated under hypoxic conditions, we analyzed the mRNA expression levels of the suspended cells for three important stem cell activation pathways (Notch, Wnt/β-catenin, and Hedgehog) downstream genes and hypoxia inducible genes (HIF1, HIF2, and ARNT). Higher mRNA expressions were observed in HIF1, HIF2, and ARNT genes in the 1% O_2_ group than in the 20% O_2_ group (Fig. 6). Furthermore, some downstream gene expression upregulation was seen: HES1 on the Notch pathway; MMP7 on the Wnt/β-catenin downstream; and PTCH1 and SMO on the Hedgehog pathway (*P* < 0.05) (Fig. 6). However, for other genes such as HES3, HES5, HEY1, HEY2, TCF-1, Gli1, and PTCH2 after qRT-PCR 50 cycles, the fluorescence amplification curves of cDNA did not reach the plateau stage. This indicates that these genes were not activated in the hematopoietic cells in our low O_2_ coculture system.

## Discussion

The physiological approach suggests that a microenvironment associating MSCs with a low O_2_ concentration would be most favorable for the maintenance of HSPCs in the course of ex-vivo expansion. The microenvironment of UCB-HSPCs in placenta or the umbilical cord is different from that in the bone marrow, and the stromal cells are dissimilar to those in bone marrow being composed of other stromal cells including WJ-MSCs and vascular endothelial cells. The microenvironment of bone marrow or cord blood is extremely hypoxic compared with ambient air [6]. The physiological low O_2_ concentrations protect the primitive hematopoietic cells against oxidative stress in vivo. Although some studies suggest that 3% O_2_ culture conditions could maintain the stemness of UCB-HSPCs better than 20% O_2_ [7, 8], our study showed some different results which might be due to our coculture system using WJ-MSCs as feeder layers and without the addition of exogenous cytokines. Furthermore, we compared a lower oxygen concentration (1%) with 20% O_2_; the results showed that, although the number of CD34^+^Lin^−^ cells was lower at 1% O_2_ than at 20% O_2_, the percentage of CD34^+^Lin^−^ cells was significant higher at 1% O_2_. Similar trends were also observed for T-CFU, CFU-GM, BFU-E, and LTC-IC in the 1% O_2_ and 20% O_2_ groups, and the LTC-IC number per 10,000 TNCs in the 1% O_2_ group was significant higher than the 20% O_2_ group, indicating that a hypoxia culture could preserve the functional capacity to maintain LTC-IC counts better than a normoxia culture. Eliasson et al. cultured mouse bone marrow HSPCs at 1% O_2_ and 20% O_2_ in suspension and found that a 1% O_2_ culture system could better maintain the stemness of HSPCs [9]. These results confirmed the major role of the microenvironment for stromal cells and low O_2_ concentrations for stem cell maintenance.

The mechanisms of how low O_2_ can maintain the stemness of HSPCs is still obscure. Some publications have already evaluated the effects of hypoxia on UCB-HSPCs. The reduction in cell division kinetics and a higher percentage of G_0_ cells in hypoxic O_2_ compared with 20% O_2_ conditions have been reported [6, 10]. Low O_2_ tension increases the expression of hypoxia-inducible factor (HIF)-1 which mediates an active switch from oxidative to glycolytic metabolism, limiting reactive oxygen species (ROS) production and promoting its degradation. ROS terminates the quiescent state of HSPCs and promotes their differentiation [11]. Recently, Mantel et al. reported that, in ambient air, HSPCs were compromised through the activation of the mitochondrial permeability transition pore; this process can be inhibited by setting a hypoxia condition during harvesting and transplantation of donor bone marrow or by using cyclosporin A, which can protect HSPCs from extraphysiologic oxygen shock/stress (EPHOSS) [12].

The mechanisms behind MSC hematopoiesis support are still elusive, whether cell-to-cell direct contact and/or soluble factor secretion [13, 14]. Proteomic analysis of the WJ-MSCs revealed high levels of interleukins (IL-1a, IL-6, IL-7, IL-8), as well as SCF, hepatocyte growth factor (HGF), and ICAM-1, suggesting once again that they may be the agents involved in the expansion of UCB-HPSCs [15]. To investigate the combination roles of WJ-MSCs with low O_2_, we assayed 10 cytokines involved in hematopoiesis: TNF-alpha, IL-6, IL-3, VEGF, SCF, IL-7, GM-CSF, M-CSF, G-CSF, and TPO in the supernatant of WJ-MSC coculture medium at 1% and 20% O_2_. The results show that the culture medium contained much higher VEGF levels in the 1% O_2_ group, and higher levels of IL-6, IL-7, SCF, and TPO in the 20% O_2_ group. It is surprising that VEGF level was so high in our coculture system at 1% O_2_, and this indicates that the hypoxic response of WJ-MSCs is characterized by a rapid increase in VEGF secretion and glycolytic activity. This rapid increase in VEGF secretion may play a critical role in the survival and expansion of human UCB-HPSCs under hypoxia. VEGF is a principal regulator of hematopoiesis, which provides for quiescence and self-renewal as well as restraining the differentiation of HSPCs [16, 17]. This may explain the reason why the TNCs harvested at 1% O_2_ contained more long-term reconstruction cells and less lineage-committed progenitor cells. The function of IL-6 and IL-7 is to induce the differentiation of HSPCs [18, 19], and our results showed that the levels of IL-6 and IL-7 were significantly higher at 20% O_2_. This may indicate a differentiation role under normoxia stimulation. Other cytokines, such as TNF-α, IL-3, M-CSF, and G-CSF which promote differentiation [20, 21], tended to decrease at 1% O_2_. This may contribute to maintaining the stemness of HSPCs. In a recent research, Paquet et al. cultured human bone marrow MSCs at different oxygen concentration (21%, 5%, and 0.1%), and showed that hypoxic conditions increased the MSC paracrine secretion of angiogenic mediators such as VEGF, IL-8, RANTES, and monocyte chemoattractant protein 1, and significantly decreased the expression of several inflammatory/immunomodulatory mediators, such as IL-6, IL-15, and IL-1Rap [22]. Majumdar et al. compared the effects of normoxia (20% O_2_) with hypoxia (2% O_2_) on the paracrine secretion of WJ-MSCs; their results showed there was significantly increased secretion of VEGF and HGF under hypoxia [23]. These studies are consistent with our findings. Taken together, MSCs exposed to a hypoxic culture increase the expression of VEGF, promote the phosphorylation of focal adhesion kinase [24], and increase the expression of chemokine receptors such as CXCR4 and CX3CR1 [25].

To investigate whether stem cell pathways were activated under hypoxic condition, we analyzed the mRNA expression levels of the harvested HSPCs on hypoxia-related genes (HIF1, HIF2, and ARNT) and Notch, Wnt/β-catenin, and Hedgehog pathway downstream genes. Our findings showed that 1% O_2_ induced higher mRNA expressions of HIF1, HIF2, and ARNT genes, and upregulated some downstream gene expression, such as HES1 on the Notch pathway, MMP7 on the Wnt/β-catenin downstream, and PTCH1 and SMO on the Hedgehog pathway. Hypoxic tension promotes the expression of the hypoxia inducible factor-related genes HIF-1α, HIF-2α, and ARNT. These genes encode HIF-α subunits that are stabilized under low oxygen tensions and exhibit tissue-restricted expression. Upon stabilization, these subunits dimerize with the β-subunit, HIF-β (ARNT), and translocate to the nucleus to regulate a spectrum of genes to maintain oxygen homeostasis, glucose metabolism, angiogenesis, erythropoiesis, and iron metabolism [26]. Hypoxia has been shown to activate molecular pathways in multiple stem cell systems. Notch signaling is widely appreciated to be critical for the maintenance of undifferentiated stem and progenitor cell populations. Ezashi et al. found that oxygen tensions as low as 1% appeared to decrease proliferation and maintain pluripotency of stem cells, while higher oxygen tensions (3–5%) appeared to maintain pluripotency with no effect on proliferation [27]. Our experiments showed that the activation of the three important signal pathways in HSPCs under hypoxia was significantly enhanced. However, the downstream genes of the three pathways were selectively activated and, thus far, their functions are not able to be shown. Some researchers have tried to confirm a correlation between HIF-related genes and stem cell pathways under hypoxia. Mukherjee et al. reported that, in drosophila blood cells, HIF-α binds to the Notch ligand intracellular segment to promote Notch downstream gene expression [28]. Bijlsma et al. found that HIF1-α induced hedgehog pathways activated by PTCH1 in the mouse [29]. Mazumdar et al. verified the relationship between HIF1 and TCF-1 in mouse embryonic stem cells [30], and Liu et al. confirmed the relationship between HIF1-α and MMP7 protein content in gastric cancer cell lines [31]. The increased expression of HIF-related factors, HES1, PTCH1, and MMP7 under hypoxia in our study can partially explain the positive effect of a hypoxic coculture system on stemness maintenance of UCB-HSPCs, but how the stem cell pathways are activated remains to be further explored.

## Conclusions

In this study, we used WJ-MSCs as a feeder layer under low O_2_ tension to simulate the physiological microenvironment in which UCB-HSPCs reside to explore the effects of the coculture system on stemness maintenance and proliferation of HSPCs in vitro without adding exogenous cytokines. The results showed that the populations of CD34^+^Lin^−^ cells and LTC-ICs could be preserved better at 1% O_2_ than at 20% O_2_. Hypoxia increased VEGF secretion and decreased IL-6 secretion and selectively activated the Notch/Wnt/Hedgehog signaling pathway in UCB-HSPCs via HIF-related factors, which plays an important role in preserving stemness and sustaining HSPC quiescence. However, our findings are only a starting point for pursuing optimizing protocols aimed at expanding UCB-HSPCs ex vivo, such as adding some key cytokines and growth factors, using optimal low O_2_ tension to protect HSPCs from EPHOSS to preserve the capacity of stemness of HSPCs, and developing bioreactor systems for in-vitro cultures, which may have important translational and clinical implications.

## Abbreviations

BFU-E: Burst-forming unit-erythroid
CFC: Colony-forming cell
CFU: Colony-forming unit
CFU-GM: Colony-forming unit-granulocyte/macrophage
EPHOSS: Extraphysiologic oxygen shock/stress
G-CSF: Granulocyte colony stimulating factor
GM-CSF: Granulocyte macrophage colony stimulating factor
HGF: Hepatocyte growth factor
HIF: Hypoxia inducible factor
HSPC: Hematopoietic stem/progenitor cell
IL: Interleukin
LTC-IC: Long-term culture-initiating cell
M-CSF: Macrophage colony stimulating factor
MNC: Mononuclear cell
qRT-PCR: Quantitative real-time polymerase chain reaction
ROS: Reactive oxygen species
SCF: Stem cell factor
T-CFU: Total colony-forming unit
TNC: Total nucleated cell
TNF: Tumor necrosis factor
TPO: Thrombopoietin
UCB: Umbilical cord blood
VEGF: Vascular endothelial growth factor
WJ-MSC: Wharton’s jelly mesenchymal stem cell
